# Microbial-mediated induced resistance: interactive effects for improving crop health

**DOI:** 10.3389/fmicb.2025.1660944

**Published:** 2025-09-22

**Authors:** Ashwini M. Charpe, Balaji Aglave, Dilip K. Ghosh

**Affiliations:** ^1^Dr. Panjabrao Deshmukh Krishi Vidyapith, Akola, Maharashtra, India; ^2^Florida Agriculture Research, Thonotosassa, FL, United States; ^3^ICAR-Central Citrus Research Institute, Nagpur, Maharashtra, India

**Keywords:** microbial mediated induced resistance, *Trichoderma*, *Bacillus*, *Pseudomonas*, oxidative burst, Ca^2+^ ion influx, secondary metabolites, context dependency

## Abstract

Microbial-mediated induced resistance (MMIR) holds great promise for sustainable agriculture, but its context dependency remains a hurdle to overcome before this potential can be realized under field conditions. MMIR is observed during interactions from the fungal biocontrol agent *Trichoderma* spp., beneficial microbes like arbuscular mycorrhizal fungi (AMFs), and bacterial species like *Bacillus* spp. and *Pseudomonas* spp., which are recognized as plant growth-promoting rhizobacteria within their plant host. Events involved in microbial induction of resistance include priming, oxidative burst, deposition of callose, Ca^2+^ ion influx, activation of transcriptional factors, activation of defense-related genes, secondary metabolite production, and regulation of stomatal activity. A defense signal cascade involves plant pathways such as the Jasmonic acid (JA) and Ethylene (ET) pathway. Reactive oxygen species (ROS) production is also triggered when plants are inoculated with these beneficial microbes. As a result, such plants become immune to future infection by pathogenic microbes. Fungi such as *Trichoderma atroviride*, *T. harzianum*, *T. longibrachiatum*, Arbuscular Mycorhizal Fungi, *Mortierella hyaline*, *Serendipita vermifera, Acrophialophora jodhpurensis, Piriformospora indica*, and bacteria *Bacillus subtilis, B. amyloliquefaciens*, *B. atrophaeus*, *B. cereus*, *B. megaterium, Paenibacillus alvei*, *Pseudomonas aeruginosa, P. fluorescens*, *Streptomyces lydicus*, *S. pactum*, and *Paraburkholderia phytofirmans* are reported to induce resistance. Work done on this aspect so far indicates that this phenomenon is highly context-dependent and is affected by biotic factors, abiotic factors, and agricultural practices. A sufficient supply of beneficial microbes in the rhizosphere is needed to induce resistance but does not guarantee triggering signal cascades if conditions are not favorable. To reduce the context dependency, it is required to simulate field-like conditions during experimentation. Alternatively, if the context dependency of MMIR is accepted as inevitable, the focus should shift to developing environmentally stable commercial formulations. Compositions of secondary metabolites from beneficial microbes, known to trigger resistance in the lab, might also induce it consistently in the field. This will require more interdisciplinary research and partnership with industries.

## 1 Introduction

Microbial-mediated induced resistance is a well-known phenomenon that has been extensively studied by scientists all over the globe due to its potential to provide eco-friendly management of crop diseases. Certain microbes have been identified to trigger the built-in resistance of plants to combat pathogen attack. The phenomenon of Induced Resistance (IR) or Induced Systemic Resistance (ISR) was first recognized by Vanpeer et al. (1991) when bacteria *P. fluorescens* strain WCS417r was reported to systemically protect carnation plants against the fungus *F. oxysporum* f. sp. *dianthi*, responsible for *Fusarium* wilt disease. They had observed the trigger of a plant-mediated resistance response in above-ground plant parts after inoculation of roots with non-pathogenic *Pseudomonas* spp. At the same time, Wei et al. (1991) reported that rhizobacterial strains protected cucumber leaves against *Colletotrichum orbiculare*, the causal agent of anthracnose disease. In order to decipher the plant-mediated protective effect they had excluded microbial antagonism by inoculating resistance-inducing rhizobacteria and the pathogens on the same plant but keeping them confined and spatially separated. The phenomenon was further observed by Gilbert et al. (1994) when certain strains of *Bacillus cereus* showed to be good biocontrol agents despite being otherwise poor colonizers. An established fungal biocontrol agent *Trichoderma* spp., beneficial microbes like arbuscular mycorrhizal fungi (AMFs), and bacterial species like *Bacillus* spp. and *Pseudomonas* spp. have been recognized as plant growth-promoting rhizobacteria and are known to induce built-in resistance in plants.

## 2 Mechanism of microbial-mediated induced resistance (MMIR)

**Figure 1:**
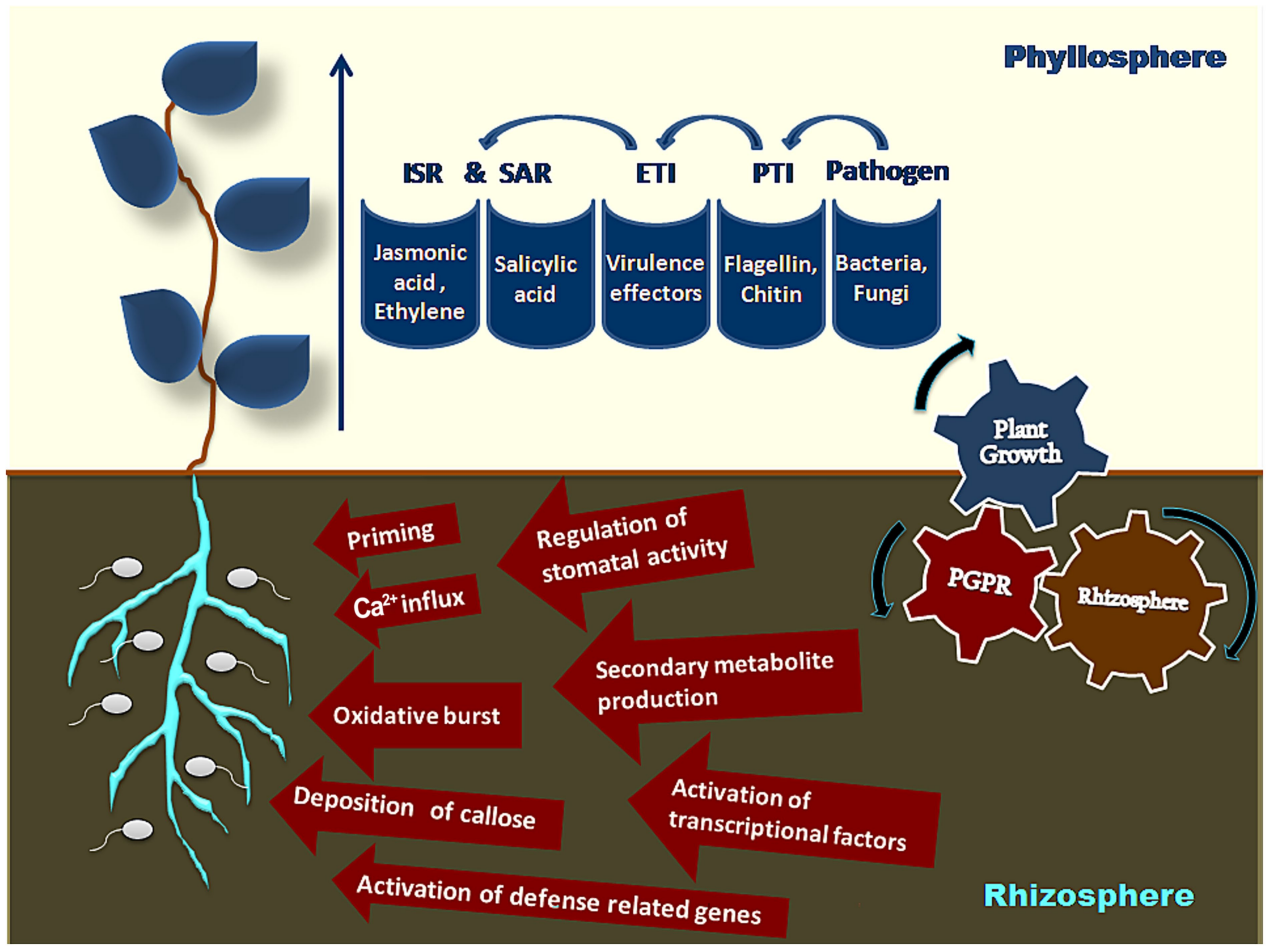
Induction of resistance by beneficial microbes.

Induced resistance triggers at two levels. The first level occurs at the time of infection, resulting in pattern-triggered immunity (PTI) due to the recognition of bacterial flagellin and fungal chitin, i.e., microbial-or pathogen-associated molecular patterns (MAMPs or PAMPs) by transmembrane pattern recognition receptors (PRRs) (Bigeard et al., 2015). This first level of defense is suppressed by the pathogen-induced virulence effectors released into plant cells by microbial secretion systems (Guo et al., 2009). The second level of immunity is triggered by these effectors and is referred to as effector-triggered immunity or ETI. These pathogen effectors are recognized by plants through nucleotide-binding leucine-rich repeat (NB-LRR) protein domains creating hypersensitive reactions to curb the pathogen attack (Jones and Dangl, 2006). Studies have also shown the involvement of PRRs in triggering ETI (Yuan et al., 2021). This intricate mechanism of immunity basically designed for host-pathogen interaction is smartly utilized by beneficial microbes to induce resistance by modulating host small RNAs to target the key elements in the process of PTI and ETI (Yu et al., 2022; Figure 1). In general, systemic resistance in plants is categorized either as induced systemic resistance (ISR) induced by non-pathogenic microbes or systemic acquired resistance (SAR) induced by pathogenic microbes. ISR is reported to operate through jasmonic acid (JA) and ethylene (ET) pathways (Pieterse et al., 1996; Knoester et al., 1999), whereas SAR operates through the accumulation of salicylic acid and thus activation of pathogenesis-related (*PR*) genes forming pathogenesis-related proteins (PR-proteins) (Gaffney et al., 1993; Van loon, 1985). However, recent reports exhibit that beneficial microbes trigger both SA and JA/ET signaling pathways to induce resistance, thus “priming” the plants for stronger and faster defense responses against the anticipated pathogen attacks (Charpe, 2019a; Charpe, 2019b; Yu et al., 2022).

Biocontrol is a significant component of plant-growth promotion by PGPR. Pathogens are contained by PGPR through the production of antibiotics (Lugtenberg and Kamilova, 2009), bacteriocins (Riley and Wertz, 2002), lytic enzymes (Neeraja et al., 2010; Maksimov et al., 2011), stress controllers (Glick et al., 2007), siderophores (Mehnaz, 2013), volatile organic compounds (VOCs), rhizospheric competence (Perez-Montano et al., 2014), ISR (Naznin et al., 2012), disrupting quorum sensing (Perez-Montano et al., 2013), competition for nutrients and niches (Kamilova et al., 2005), and hyperparasitism (Harman et al., 2004; Kamilova et al., 2008) (Figure 1). Pathogens are restricted by PGPRs by one or a few of these mechanisms.

ISR was first described by Vanpeer et al. (1991) in carnation plants systemically protected by *Pseudomonas fluorescens* strain WCS417r against *Fusarium* wilt caused by *Fusarium oxysporum* f. sp. *dianthi* and by Wei et al. (1991), who reported that inoculation of cucumber roots with non-pathogenic *Pseudomonas* spp. protected leaves from anthracnose caused by *Colletotrichum orbiculare*. A similar phenomenon was recorded by Gilbert et al. (1994) with *B. cereus*. Signaling molecules accumulated in plants due to exogenous application of non-pathogenic *Pseudomonas* and *Bacillus* spp. are shown to trigger ISR (Ryals et al., 1996; Van Loon et al., 1998).

Rhizobacteria-mediated ISR and pathogen-induced SAR both make uninfected plant parts more resistant to plant pathogens (Van Wees et al., 1997; Van Loon et al., 1998) i.e., fungi, bacteria, virus, nematodes, and insects (Zehnder et al., 1997; Van Loon et al., 1998; Bent, 2006; Pozo and Azcon-Aguilar, 2007). Rhizobacteria-mediated ISR is demonstrated in several species of plants like bean, tomato, tobacco, radish, cucumber, and carnation (Van Loon et al., 1998), depending on the specificity of the interaction between plants and rhizobacteria (Van Loon, 2007). It is noted that the same strain triggers ISR against several pathogens in the same plant (Somers et al., 2004). Whereas a PGPR that triggers ISR in one plant species may not trigger ISR in another (Vleesschauwer and Hofte, 2009).

ISR does not require extensive colonization of the root system, as observed in the case of *Pseudomonas fluorescens* WCS365 (Dekkers et al., 2000). It is also explained that dependency of ISR on JA and ET is based on enhanced sensitivity to these hormones and not on an increase in their production (Pieterse et al., 2000, 2001). At the same time, ISR is found to impart less protection than SAR (Van Loon, 2000) and also depends on plant genotype (Bloemberg and Lugtenberg, 2001). However, ISR and SAR together provide better protection than alone, justifying their additive effect (Van Wees et al., 2000).

Specifically, *Pseudomonas, Bacillus*, and *Azospirillum* genera are the major group of PGPRs that trigger ISR (Kloepper et al., 2004; Van Wees et al., 2008). A few other species of symbiotic rhizobacteria used as a coinoculant with different PGPRs have also shown ISR activity (Elbadry et al., 2006).

ISR and SAR, which are part of plants’ systemic resistance responses, are activated by certain microorganism molecules known as elicitors. Cell wall polysaccharides (lipopolysaccharides (LPS) and exopolysaccharides (EPS)) are the most described biotic elicitors, along with flagella, salicylic acid, cyclic lipopeptides, antifungal factor Phl, siderophores, antibiotics such as 2,4-diacetylphloroglucinol, the signal molecules AHL, biosurfactants, N-alkylated benzylamines, and volatile blends and individual volatiles of acetoin and 2,3-butanediol produced by *B. subtilis* GB03 (Ryu et al., 2003; Iavicoli et al., 2003; Shuhegge et al., 2006; Ongena et al., 2007; Van Loon, 2007; Ramos et al., 2008; Berg, 2009; Vleesschauwer and Hofte, 2009; Doornbos et al., 2012).

Further, the JA signaling pathway is reported to be controlled by two branches of regulators, the MYC branch and the ERF branch. During wound stress and necrotrophic pathogen attack, JA signaling is regulated by the MYC-type transcriptional regulator and APETALA2/ethylene response factor (AP2/ERF) family, such as ERF1 and ORA59 (Lorenzo et al., 2003).

The application of *Trichoderma atroviride* fungus results in the transcriptional regulators of *Arabidopsis thaliana*, i.e., the WRKY genes of the SA pathway, differentially expressing in a time-dependent manner. At the same time, positive regulators of the JA pathway such as AtWRKY8 and AtWRKY33 were also found to be expressed (Saenz-Mata et al., 2014).

The application of the *B. cereus* strain AR156-triggered ISR to Arabidopsis resulted in the involvement of WRKY11 and WRKY70 through the JA and SA signaling pathways, respectively (Jiang et al., 2016a,b).

In the case of beneficial *Pseudomonas fluorescens* WCS417r, the transcriptional regulator MYB72 was activated upon colonization and was required in the early signaling steps of microbe-mediated ISR, acting upstream of ethylene in the signaling pathway (Van der Ent et al., 2008).

Rudrappa et al. (2008a,b) reported that infection of *A. thaliana* seedling leaves with the foliar pathogen *P. syringae* pv. *tomato* Pst DC3000 resulted in enhanced secretion of l-malic acid by the roots. Chemotaxis selectively recruits the beneficial *B. subtilis* FB17 strain and protects the plant through ISR. The biocontrol bacterium *P. fluorescens* WCS365 has also demonstrated strong chemotaxis towards the major tomato root exudate component through citric acid (DeWeert et al., 2002) that also acts through ISR (Kamilova et al., 2005).

Both ISR and SAR can overlap in some cases (Jiang et al., 2016a,b). In many cases, SAR can also be triggered without tissue necrosis, as demonstrated in *Arabidopsis thaliana* (Mishina and Zeier, 2007). Biopriming plants with some PGPRs can also provide systemic resistance against a broad spectrum of plant pathogens.

## 3 Events involved in MMIR

To begin with, the ‘priming’ of plants by beneficial microbes activates defense mechanisms, leading to an oxidative burst, callose deposition, Ca^2+^ influx, production of transcription factors, activation of defense-related genes, synthesis of secondary metabolites, and regulation of stomatal activity (Yu et al., 2022). These shall now be discussed individually.

### 3.1 Priming

Beneficial microbes produce certain ligands like flagellin, lipopolysaccharides (LPS), exopolysaccharides, and chitin oligosaccharides (Zhang and Zhou, 2010; Zamioudis and Pieterse, 2012; Zipfel and Oldroyd, 2017; Saijo et al., 2018) that are recognized by receptor proteins of plants that transfer the signals to co-receptors. Subsequent reactions involve the phosphorylation of the downstream substrates, producing a signal cascade resulting in an oxidative burst, Ca^2+^ influx, MAP-Kinase activation, and hormone signaling activation (Bazin et al., 2020). In a wide array of eubacteria, the N-terminal part of flagellin is found to be highly conserved with 22-amino acid epitope, known as flg22 (Felix et al., 1999). The first receptor that is reported to recognize flagellin of PGPRs is the FLAGELLIN-SENSING 2 (FLS2) receptor (Trda et al., 2014). It is reported that recognition of flg22 by FLS2 initiates its heterodimerization with the co-receptors BRI1-associated kinase (BAK1) and BAK1-LIKE1 (BKK1) that phosphorylate the receptor-like cytoplasmic kinase *Botrytis*-induced kinase1 (BIK1), thus starting PTI signaling (Chinchilla et al., 2007; Lu et al., 2010; Segonzac and Zipfel, 2011). This was demonstrated by the experiments conducted with *Arabidopsis thaliana* (Lu et al., 2010).

In the case of plant symbiosis with rhizobia and AMF, the process is triggered by chitin-derived oligosaccharide signals (Zipfel and Oldroyd, 2017). In this example, acylated lipo-chitooligosaccharides known as Nod factors are produced by rhizobia that are recognized by LysM receptor-like kinases that activate common symbiotic pathways controlling both mycorrhization by AFMs and nodulation by rhizobia (Madsen et al., 2003; Gough and Cullimore, 2011; Yu et al., 2022).

PTI induced by beneficial microbes are found to be transient and relatively mild as compared to the pathogen-induced PTI that causes severe cellular damage resulting in mutually beneficial interaction with the plant. Felix et al. (1999) has reported that in the example of flg22 peptide obtained from *Burkholderia phytofirmans*, only small oxidative bursts sufficient enough to induce defense genes was observed. Similarly, in the case of *P. fluorescens* WCS417, low molecular compounds were produced that were able to suppress flagellin-triggered PTI responses in Arabidopsis roots (Millet et al., 2010). Both these examples highlight the underlying processes involved in co-evolution without the beneficial microbes getting harmed by the defense response. Furthermore, beneficial microorganisms can induce different pathways by triggering various defense responses of host plants and imparting resistance to multiple pathogens. Through such versatile ISR, *Bacillus amyloliquefaciens*, *B. atrophaeus*, *B. cereus*, and *Pseudomonas fluorescens* were demonstrated to be effective against fungal, bacterial, and viral infections. Such priming by the beneficial microbes not only induced early plant ISR events but also increased the expression of pathogenesis-related *PR-*genes and the activities of defense-related substances, such as phenylalanine ammonia-lyase, polyphenol oxidase, peroxidase, β-1,3 glucanase, and chitinase. The accumulation of reactive oxygen species was also reported to be enhanced (Guo et al., 2019; Wang et al., 2020).

### 3.2 Oxidative burst

Oxidative burst is represented by the production of a large number of reactive oxygen species (ROS), including superoxide anion (O^2−^), hydroxyl radical (OH.), and hydrogen peroxide (H_2_O_2_), by plants under stressed conditions (Chen et al., 1993). The induction of oxidative bursts ultimately results in an immune response toward pathogens, leading to programmed cell death and stomatal closure (Apel and Hirt, 2004). Since the accumulation of ROS also causes damage to plant tissues (Dat et al., 2000), it is required to control the production of ROS by enzymatic and non-enzymatic reactions. Enzymes like peroxidase (POX), polyphenol oxidase (PPO), superoxide dismutase (SOD), ascorbate peroxidase (APX), glutathione peroxidase (GPX), and catalase (CAT) help to control ROS production by reducing superoxide to H_2_O (Yu et al., 2022). Production of ROS is reported in *Bacillus cereus* and *Pseudomonas aeruginosa*.

### 3.3 Deposition of callose

When a plant is attacked by a pathogen, callose, a β-1,3-glucan polymer, accumulates in the cell wall at the infection site, thickening it to restrict fungal germ tube invasion. Clay et al. (2009) explained the significance of *PEN2* and *PEN3* genes required for callose deposition and consequently for pathogen resistance. Sakthivel and Balachandar (2019) reported that the MAMPs released by PGPR generate ROS and increase the level of SA. High levels of SA regulate the PDLP5-dependent expression of the callose synthase gene (CALS10), triggering callose deposition by the plant.

### 3.4 Ca^2+^ ion influx

Microbial elicitors are known to trigger ion fluxes like Ca^2+^ influx, Cl^−^ effluxes, and K^+^/H^+^ exchange. These ion influxes are important for the development of cells, immunity of the plants, and transportation of signals. Ca^2+^ ion influx is the most important ion influx, playing a significant role as a secondary messenger in diverse cellular processes and various physiological changes (Trewavas and Malho, 1998). Ca^2+^ ion influx induced by microbial elicitors not only acts as a mediator in events but, through Ca^2+^-dependent H_2_O_2_ production, Ca^2+^ signaling is amplified and increases Ca^2+^ ion influx from extracellular sources (Price et al., 1994; Lecourieux et al., 2002). Pretreatment with forskolin, dibutyryl cAMP, or Ca^2+^ ionophore A23187 is reported to enhance the production of ROS, thus restricting infection from *Colletotrichum lindemuthianum* in bean (*Phaseolus vulgaris*). Further, in a cross check, treatment with the Ca^2+^ channel blocker was found to decrease the oxidative burst, highlighting the role of Ca^2+^ influx in ROS production (Bindschedler et al., 2001). It is reported that, after Ca^2+^ ion influx, a Ca^2+^ ion sensor calmodulin is activated due to the binding of Ca^2+^ ions further activating protein phosphatase and Ca^2+^/calmodulin-dependent protein kinase (CDPK), membrane-bound enzymes, or transcription factors, thus regulating transcription in plants (Zhao et al., 2005; Iqbal et al., 2020). CDPK plays an important role in the defense responses of plants. Protein kinase cascades induced by Ca^2+^ spiking play a role in the production of ROS, transfers lipid signaling messengers, and amplifies the elicitor signals to downstream reactions. Ca^2+^ spiking also differentially activates transcription factors, directly regulating extensive defense gene expression (Dolmetsch et al., 1997; Yang and Poovaiah, 2002; Iqbal et al., 2020). Ca^2+^/calmodulin-binding transcription factors modulate EDS1 to regulate salicylic acid levels in plant cells (Du et al., 2009).

### 3.5 Activation of transcriptional factors

In the JA/ET signaling pathway, several transcription factors play a crucial role in regulating the induction of resistance. WRKY transcription factors are reported to differentially express during beneficial plant–microbe interactions (Saenz-Mata et al., 2014). The MYB family proteins that regulate plant development are also found to regulate plant–microbe interactions. MYC2, a basic helix–loop–helix (bHLH) transcription factor, is found to be involved in IR triggered by beneficial microbes (Dubos et al., 2010; Kazan and Manners, 2013). Ethylene response factor1 (ERF1) is a transcription factor that regulates the expression of pathogen response genes to prevent disease progression and is found to be functional during beneficial microbe-plant interaction. In both JA and ET signaling pathways, the expression of ERF1 is activated rapidly and synergistically (Lorenzo et al., 2003).

### 3.6 Activation of defense-related genes

The induction of SA and JA/ET pathways during beneficial microbe-plant interactions is the key to activation of resistance genes to combat pathogen attack. Effective use of defense mechanisms of microbial-induced resistance depends on an accurate and context-specific regulation of gene expressions. This needs an understanding of complex circuits and regulatory networks due to interactions between genes and their products. In a study conducted by Timmermann et al. (2020), regulatory mechanisms of the induced resistance triggered by the beneficial bacterium *Paraburkholderia phytofirmans* PsJN was explored and a regulatory network according to gene expression and time series data was drawn. Pre-treatment of *Arabidopsis thaliana* with the non-pathogenic *Bacillus cereus* AR156 strain was found to trigger the expression of *PR1*, *PR2*, and *PR5* genes and Plant Defensin 1.2 (PDF1.2) accumulation; this indicates the activation of SA and JA/ET signaling pathways (Niu et al., 2011; Niu et al., 2016a,b; Nie et al., 2017). *NPR1* is reported to coordinate SA and JA signaling pathways, regulating downstream defense response genes (Cao et al., 1994; Cao et al., 1997; Pieterse et al., 1998; Spoel et al., 2003).

### 3.7 Secondary metabolite production

Secondary metabolites produced by the plants help them to adapt to various stresses under natural conditions. Interaction of these secondary metabolites with beneficial microorganisms can modulate plant growth and immune responses, thus inhibiting metabolism and/or growth of harmful microbes. For example, selective growth of PGPRs in the plant rhizosphere is controlled by root exudates enhancing biofilm formation of beneficial microbes (Zhang et al., 2014). A list of plant metabolites reported to play significant roles in beneficial microbe-plant interactions is given here (Table 1).

**Table 1:** Secondary metabolites of plants reported to regulate beneficial microbe-plant interactions.

SN	Secondary plant metabolite	Plant	Beneficial microbe	Mode of action	Reference
1	L-malic acid (L-MA) (root exudates)	*—*	PGPR *Bacillus subtilis* FB17	Promotes selective growth of beneficial rhizobacteria	Rudrappa et al. (2008a,b)
2	7,40-dihydroxyflavone (Flavonoid)	*Medicago sativa*	Acidobacteria, Gaiellales, Nocardioidaceae and Thermomonosporaceae	Controls relative abundance of beneficial microbes in root zone	Szoboszlay et al. (2016)
3	Luteolin (Flavonoid)	Leguminous plants	Rhizobium	Work as signaling molecule to initiate symbiosis	Abdel-Lateif et al. (2012)
4	Strigolactones (Plant Harmone)	—	Arbuscular Mycorrhiza Fungi (AMF)	Stimulates branching of fungal hyphae of arbuscular mycorrhiza	Al-Babili and Bouwmeester (2015)
5	Camalexin	*Arabidopsis*	PGPR *Pseudomonas fluorescens* SS101	Regulates SA signaling-dependent resistance	Van de Mortel et al. (2012)
6	Glucosinolates	*Arabidopsis*	PGPR *Pseudomonas fluorescens* SS101	Regulates SA signaling-dependent resistance	Van de Mortel et al. (2012)

Secondary metabolites produced by the beneficial microbes are found to be antagonistic to the pathogen and are reported as elicitors of immune response to induce resistance in plants (Prsic and Ongena, 2020). Some significant secondary metabolites produced by beneficial microbes are listed in Table 2.

**Table 2:** Antagonistic secondary metabolites reported to be produced by beneficial microbes and their mode of action.

SN	Secondary metabolites	Beneficial microbe	Mode of action	Reference
1	Phenazines	*Pseudomonas*	Antifungal activity and were able to elicit ISR	Chin-A-Woeng et al. (2003)
2	Extracellular polysaccharides (EPS)	*B. cereus* AR156	Induces systemic resistance to Pst DC3000 in Arabidopsis	Jiang et al. (2016a,b)
3	Lipopolysaccharides (LPS)	—	Trigger the activation of signal transduction pathways involved in phytohormones SA and JA, and the associated methyl esters and sugar conjugates	Finnegan et al. (2016)
4	Harzianic acid	*Trichoderma harzianum* M10	Modulates the signaling pathway and differentially expressed genes (DEGs) involving JA/ET-and SA-mediated signaling pathways and increased reactive oxygen species (ROS)	Manganiello et al. (2018)
5	Microbial volatile compounds (MVCs)	—	Promotes plant growth via improved photosynthesis rates, enhances immune system, and activates phytohormone signaling pathways	Kong et al. (2018)
6	Volatile Organic Compounds (VOCs)	—	Affects ISR and their interactions with SA, JA/ET, and auxin signaling pathways	Tyagi et al. (2018), Garbeva and Weisskopf (2020) and Cellini et al. (2021)
7	VOC 2,3-butanediol,	*Bacillus* spp.	Elicitors of ISR	Ryu et al. (2004) and Chowdhury et al. (2015)
8	Cyclic lipopeptides surfactin	*Bacillus* spp.	Elicitors of ISR	Ryu et al. (2004) and Chowdhury et al. (2015)

These findings demonstrate the intricate framework of secondary metabolites produced by plants to support beneficial microbes and restrict harmful ones, while also enhancing plant resistance through secondary metabolites generated by beneficial microbes, ultimately protecting the plant from pathogen attack.

### 3.8 Regulation of stomatal activity

Photosynthesis, respiration, and transpiration are the most important physiological activities of plants regulated by the stomata. Melotto et al. (2006) has observed that, to restrict the entry of pathogenic bacteria, the plant closes its stomatal openings, resulting in reduced gaseous exchange and thus reduced photosynthesis. Abscisic acid (ABA) produced by plants under stressed conditions is demonstrated to regulate stomatal opening. ABA mediates stomatal closure through three steps of signal transduction. In the first step, ABA binds to ABA cell receptors and interacts with PP2C, a group of type 2C protein phosphatases (Park et al., 2009; Ma et al., 2009). In the second step, this binding results in the inactivation of the inhibitory regulatory function of PP2C and the activation of SnRK2 protein kinase OST1 (Umezawa et al., 2009). In the third step, thus activated, OST1 directly binds and phosphorylates to activate the Slow Anion Channel-Associated1 (SLAC1) anion channel that mediates anion release from the guard cells. Stomatal closure then takes place (Geiger et al., 2009; Lee et al., 2009; Brandt et al., 2012). Through other routes, OST1 can catalyze hydrogen peroxide (H_2_O_2_) production (Sirichandra et al., 2009; Raghavendra et al., 2010) and the produced H_2_O_2_ modulates ABA signaling in the plasma membranes of guard cells (Pei et al., 2000) by activation of calcium channels. Lipoxygenase-encoding gene *LOX1* is another signaling component that coordinates stomatal regulation. It is a JA-responsive gene that triggers stomatal defense by expressing in guard cells in response to PAMPs. This indicates that the JA signaling pathway also participates in regulating stomatal defense (Montillet et al., 2013). The triggering of ABA and JA pathways is demonstrated by PGPR *B. amyloliquefaciens* FZB42 by production of acetoin and 2,3-butanediol that induces the closing of stomata in response to pathogen attack (Wu et al., 2018a; Wu et al., 2018b; Xie et al., 2018). This indicates coordination of multiple signaling components to regulate microbial-mediated stomatal defense.

## 4 Microbial induction of resistance in plants

Various species of fungi and bacteria that are beneficial to plant growth are reported to trigger the innate resistance of plants and help them combat pathogen attack. The role of these fungi and bacteria in induction of resistance is discussed here.

### 4.1 Fungi-mediated IR

Beneficial fungi, such as *Trichoderma* spp. and AMF, are known to induce resistance to biotic stresses in plants through various mechanisms. Here, we will discuss the role of different beneficial fungi in modulating plant defense.

#### 4.1.1 Trichoderma-mediated IR

##### 4.1.1.1 Trichoderma atroviride

Glutamate glyoxylate amino transferase GGAT1 is responsible for the stimulation of plant growth and induction of the plant systemic resistance. WRKY transcription factors mediate active defense response to biotic and abiotic stresses and are triggered by *T. atroviride*, resulting in the induction of resistance to *Botrytis cinerea* in *Arabidopsis thaliana* (Saenz-Mata et al., 2014; Gonzalez-Lopez et al., 2021).

##### 4.1.1.2 Trichoderma harzianum

*T. harzianum* is reported to induce resistance to spot blotch disease caused by *Bipolaris sorokiniana* in bread wheat (*Triticum aestivum* L.) by triggering the methyl jasmonate pathway, resulting in enhanced phenylpropanoid activities that decrease tissue disintegration and cell wall disruption and increase lignification and suberization of the plant cell (Singh et al., 2019). Similarly, the response of tomato to the wilt-causing pathogen *Rhizoctonia solani* is reported to be modulated by *T. harzianum* and its secondary metabolite harzianic acid. Harzianic acid modulates the signaling pathway and differentially expressed genes (DEGs) involving JA/ET-and SA-mediated signaling pathways and increases reactive oxygen species (ROS) (Manganiello et al., 2018). Thus, it induces the expression of several defense response-related genes. Further, *T. harzianum* OTPB3 is reported to stimulate growth and induce systemic resistance in tomato against early blight disease incited by *Alternaria solani* and late blight disease incited by *Phytophthora infestans* mediated by the production of defense-related enzymes viz. peroxidase, polyphenol oxidase, and superoxide dismutase that inhibit mycelial growth and spore germination of pathogens and protect the plant from oxidative stress (Chowdappa et al., 2013). *T. harzianum* T-203 is reported to trigger defense responses in cucumber plants (*Cucumis sativus* L.) by increasing the chitinase and peroxidase activities and forming callose barriers to restrict the entry of pathogens (Yedidia et al., 1999). Bigirimana et al. (1997) has reported the induction of systemic resistance by *T. harzianum* in common bean (*Phaseolus vulgaris*).

##### 4.1.1.3 Trichoderma longibrachiatum

*Trichoderma longibrachiatum* MK1 is reported to restrict *Botrytis cinerea*, *Alternaria alternata*, *Pythium ultimum*, and *Rhizoctonia solani* pathogens by producing type II hydrophobin that is antifungal and a plant growth promoter (PGP) (Ruocco et al., 2015).

### 4.2 Arbuscular mycorhizal fungi-mediated IR

The beneficial root-colonizing fungi known as Arbuscular Mycorhizal Fungi (AMF) is also reported to trigger the immune response of plants to pathogen attack.

#### 4.2.1 Mortierella hyalina

This root-colonizing endophytic fungus promotes the growth of aerial parts of the *Arabidopsis thaliana* plant but not the roots. Fungal exudates are recorded to induce transient cytoplasmic Ca_2+_ elevation in the roots that restrict *Alternaria brassicae* infection (Johnson et al., 2019). The Ca_2+_ response did not require the well-characterized (co) receptors BAK1, CERK1, or FLS2 for pathogen-associated molecular patterns or the Ca_2+_ channels GLR-2.4, GLR-2.5, and GLR-3.3 or the vacuolar TWO PORE CHANNEL1, which are usually involved in cytoplasmic Ca_2+_ elevation. Ca_2+_ is known to regulate the permeability of plant cell membranes to enhance resistance. This interaction also triggers the Jasmonic acid pathway that induces plant resistance to abiotic and biotic stresses.

#### 4.2.2 Serendipita vermifera

This fungal root endophyte exhibits inter-kingdom synergistic effects with the microbiota in *Arabidopsis thaliana* and barley (Sarkar et al., 2019). *Serendipita vermifera* is reported to synergistically impart resistance in collaboration with soil bacteria against the soil-borne pathogen *Bipolaris sorokiniana* of *A. thaliana* and Barley. On the basis of RNA-sequencing, they showed that these beneficial activities were not associated with extensive host transcriptional reprogramming but rather with the modulation of expression of microbial effectors and carbohydrate-active enzymes (Mahdi et al., 2022). It was observed to trigger the production of ROS, causing inhibition of the mycelial growth and spore germination and activation of hydrolytic enzymes, resulting in the activation of defense.

#### 4.2.3 Acrophialophora jodhpurensis

This endophyte is reported to have direct antagonistic activity and induce resistance to *Rhizoctonia solani* AG4-HGII, a fungal pathogen responsible for root rot and crown rot diseases in Tomato. Apart from direct antagonism, the endophyte also triggers ROS production, resulting in inhibition of the mycelial growth and spore germination, activation of the defense enzymes peroxidase, chitinase, and beta-1,3-glucanase, and inhibition of mycelial growth, spore germination, and phenyl alanine ammonia lyase that regulate plant growth and stress tolerance. It also restricts iron, thus inhibiting pathogen growth and promoting plant growth (Daroodi and Taheri, 2021).

Isolate Msh5 of the endophyte is reported to promote tomato plant growth and control *Alternaria alternata*, the causal agent of early blight in tomatoes (Daroodi et al., 2022). In this study, morphological and molecular analyses based on ITS and tub2 sequences revealed that the fungal isolate, Msh5, was *Acrophialophora jodhpurensis* (*Chaetomium jodhpurense* Lodha). This endophyte was capable of producing indole-3-acetic acid (IAA), urease, siderophore, and extracellular enzymes and could solubilize phosphate. The Msh5 isolate of *A. jodhpurensis* inhibited *A. alternata* growth in dual culture, volatile, and non-volatile metabolites assays *in vitro*. The supernatant of this endophytic fungus reduced the spore germination and altered the hyphal structure of *A. alternata*. At the same time, the germ tubes produced by spores had vacuolization and abnormal morphology as compared to control. *In vivo* studies also revealed significant increases in plant-growth parameters of tomato plant and reduced disease progression of *A. alternata*, proving it as a potential biofertilizer and biocontrol agent against *A. alternata*.

#### 4.2.4 Piriformospora indica

*Piriformospora indica* is a growth-promoting root endosymbiont. Its cell wall extract was found to transiently alleviate cytosolic Ca^2+^ in Arabidopsis and tobacco through activating CYCLIC NUCLEOTIDE GATED CHANNEL 19 (CNGC19), an important Ca^2+^ channel that affects mutualistic interaction with the plants (Vadassery et al., 2009; Jogawat et al., 2020).

### 4.3 Bacteria-mediated IR

Many bacterial genera and species are reported as potential PGPRs and biocontrol agents. They will be discussed here individually.

#### 4.3.1 Bacillus subtilis

Many strains of *B. subtilis* are reported to induce resistance in plants. According to studies by Lakshmanan et al. (2013), *Bacillus subtilis* FB17 was found to confer resistance to *Pseudomonas syringae* pv. tomato (Pst) DC3000, mediated by malate efflux that enabled stable colonization. Bigirimana et al. (1997) reported induction of resistance by *B. subtilis* M4 against *Colletotrichum lagenarium* and *Pythium aphanidermatum* due to metabolic and transcriptomic changes, resulting in an enhanced defense response. *Bacillus subtilis OTPB1* was reported to impart resistance by Chowdappa et al. (2013) to *Alternaria solani* and *Phytophthora infestans* responsible for early and late blight of tomato, respectively, due to activation of defense-related enzymes viz. peroxidase, polyphenol oxidase, and superoxide dismutase, resulting in inhibition of mycelial growth and spore germination and protection from oxidative stress. Another strain, *B. subtilis* UMAF6639, showed the induction of resistance against *Podosphaera fusca*, which causes powdery mildew of cucurbits, by stimulating the production of reactive oxygen species, resulting in inhibition of mycelial growth and spore germination. It also caused cell wall reinforcement, which resulted in a reduction in pathogen invasion and the production of metabolites like surfactin lipopeptides, resulting in the stimulation of the immune response (Garcia-Gutierrez et al., 2013).

#### 4.3.2 Bacillus amyloliquefaciens

*Bacillus amyloliquefaciens* Ba13 was found to induce resistance to tomato yellow leaf curl virus by activating *PR1*, *PR2*, and *PR3* genes, which have antimicrobial effects due to enhanced phenylalanine ammonia lyase, beta-1,3 glucanase, and chitinase activities. Enhanced activities of phenylalanine ammonia lyase resulted in the regulation of plant growth and stress tolerance. Beta-1,3 glucanase caused inhibition of mycelial growth and spore germination, and chitinase inhibited mycelial growth (Guo et al., 2019). Another strain, *B. amyloliquefaciens* FZB42, was reported to induce resistance against *Phytophthora nicotianae* and *Rhizoctonia solani* which cause leaf blight disease in *Nicotiana benthamiana* and bottom rot in lettuce, respectively, mediated by ABA/SA-induced stomatal closure, resulting in a reduction in pathogen invasion. It also resulted in activation of the defense-related genes*PR-la*, *LOX*, and *ERF1* and the production of secondary metabolites viz. surfactin, fengycin, and bacillomycin D that resulted in a direct antagonistic effect and induction of defense-related genes (Chowdhury et al., 2015; Wu et al., 2018a,b).

#### 4.3.3 Bacillus atrophaeus

Ayaz et al. (2021) reported the induction of resistance to the root-knot nematode *Meloidogyne incognita* by *B. atrophaeus* GBSC56 due to the production of volatiles like dimethyl disulfide, methyl isovalerate, and 2-undecanone as well as the regulation of antioxidant enzymes and protection from oxidative stress and the antagonistic effect on *M. incognita* in tomato.

#### 4.3.4 Bacillus cereus

*Bacillus cereus* AR156 was reported to induce resistance against *Pseudomonas syringae* pv. tomato (Pst) DC3000 by suppressing miR825 and miR825, thereby activating the targeted defense-related genes in *Arabidopsis thaliana* (Niu et al., 2016a,b; Nie et al., 2019). Another strain, *B. cereus* C1L, was reported by Huang et al. (2012) to induce resistance against *Botrytis cinerea* and *Cochliobolus heterostrophus*, which are responsible for foliar and soil diseases, by the production of a volatile metabolite dimethyl disulfide, which is an elicitor for the induction of ISR.

#### 4.3.5 Bacillus megaterium

Chakraborty et al. (2006) reported the induction of resistance in *Camellia sinensis* when treated with *Bacillus megaterium* DE BABY TRS-4 against brown root rot caused by *Fomes lamaoensis* due to the enhanced activity of enzymes viz. peroxidase, chitinase, and beta-1,3-glucanase responsible for the inhibition of mycelial growth and spore germination. Phenyl alanine ammonia lyase was responsible for the regulation of plant growth and stress tolerance. Enhanced phosphate solubilization and production of IAA resulted in the promotion of plant growth and the regulation of siderophore and antifungal metabolites resulted in the inhibition of pathogen growth.

#### 4.3.6 Paenibacillus alvei

Tjamos et al. (2005) found *Paenibacillus alvei* K165 was able to induce defense-related *PR-1*, *PR2*, and *PR-5* genes, which have antimicrobial effects, as well as beta-1,3 glucanase and chitinase activities, which are markers for SA-mediated activation of SAR against *Verticillium dahlia* in *A. thaliana*.

#### 4.3.7 Pseudomonas aeruginosa

*Pseudomonas aeruginosa* 7NSK2 was reported by De Vleesschauwer and Hoefte (2006) and De Meyer et al. (1999) to induce resistance against Rice blast and sheath blight diseases caused by *Magnaporthe grisea*, *Rhizoctonia solani* respectively by producing metabolites viz. phenazine, pyocyanin and pyochelin that result in the induction of ISR. The production of ROS results in the inhibition of mycelial growth and spore germination, and the production of SA results in the expression of acquired resistance.

#### 4.3.8 Pseudomonas fluorescens

Vanpeer et al. (1991) reported *P. fluorescens* strain WCS417r to systemically protect carnation plants against the fungus *F. oxysporum* f. sp. *dianthi*, responsible for *Fusarium* wilt disease. Van de Mortel et al. (2012) has reported metabolic and transcriptomic changes resulting in the induction of resistance responses by *Pseudomonas fluorescens* SS101 against *Pseudomonas syringae* pv tomato (Pst) in *Arabidopsis thaliana* (Van de Mortel et al., 2012). Another strain, *P. fluorescens* PTA-CT2, was found to induce resistance to *Plasmopara viticola* and *Botrytis cinerea*, which cause downey mildew and gray mold diseases in grapes, respectively, by the activation of SA, JA, and ABA defensive pathways, resulting in a reduction in pathogen invasion (Lakkis et al., 2019). Further, *P. fluorescens* WCS417 was reported to induce broad spectrum resistance by activation of the transcription factor MYB72 responsible for the regulation of iron-uptake responses (Vanpeer et al., 1991).

#### 4.3.9 Streptomyces lydicus

*Streptomyces lydicus* M01 was found to induce resistance against *Alternaria alternata*, which causes foliar disease of cucumbers, by inducing production of ROS that results in the inhibition of mycelial growth and spore germination (Morcillo et al., 2020).

#### 4.3.10 Streptomyces pactum

This is another bacterium responsible for inducing resistance to tomato yellow leaf curl virus as reported by Li et al. (2019). In this case, resistance is induced through multiple routes, such as the production of ROS, which inhibits mycelial growth and spore germination; activation of enzymes such as peroxidase, chitinase, and β-1,3-glucanase, which inhibit mycelial growth and spore germination, and phenylalanine ammonia-lyase, which regulates plant growth and stress tolerance; activation of defense-related genes *PR-1*, *PR-2*, and *PR-5*, which exert antimicrobial effects by activating β-1,3-glucanase and chitinase as markers of SA-mediated SAR; and JA/ET-mediated induction of immune responses, which reduces pathogen invasion.

#### 4.3.11 Paraburkholderia phytofirmans

*Paraburkholderia phytofirmans* PsJN is a beneficial endophytic bacteria able to colonize a wide range of plants. In addition to its ability to promote plant growth, this endophytic bacteria is capable of inducing resistance against biotic as well as abiotic stresses in various plants (Esmaeel et al., 2018).

A crop-wise summary of various microorganisms responsible for induction of resistance in plants is presented in Tables 3–7.

**Table 3:** Microorganisms that have exhibited induction of resistance in the model plant *Arabidopsis thaliana*.

SN	Crop	Microorganism responsible for IR	Plant disease/pathogen	Reference
1	Arabidopsis (*Arabidopsis thaliana* L.)	*Paenibacillus alvei* K165	*Verticillium dahlia* in *A. thaliana*.	Tjamos et al. (2005)
		*Pseudomonas fluorescens* SS101	*Pseudomonas syringae* pv tomato (Pst)	Van de Mortel et al. (2012)
		*Bacillus cereus* AR156	*Pseudomonas syringae* pv. tomato (Pst) DC3000	Niu et al. (2016a,b) and Nie et al. (2019)
		*Mortierella hyalina*	*Alternaria brassicae* infection in roots	Johnson et al. (2019)
		*Serendipita vermifera*	Soil borne pathogen *Bipolaris sorokiniana*	Sarkar et al. (2019)
		*Piriformospora indica*	—	Vadassery et al. (2009) and Jogawat et al. (2020)
		*Trichoderma atroviride*	*Botrytis cinerea*	Saenz-Mata et al. (2014) and Gonzalez-Lopez et al. (2021)

**Table 4:** Microorganisms that have exhibited induction of resistance in Tomato (*Solanum lycopersicum* L.).

SN	Crop	Microorganism responsible for IR	Plant disease/pathogen	Reference
1	Tomato (*Solanum lycopersicum* L.)	*B. subtilis* M4	*Pythium aphanidermatum*	Bigirimana et al. (1997)
		*T. harzianum* OTPB3	Early blight disease incited by *Alternaria solani* and late blight disease incited by *Phytophthora infestans*	Chowdappa et al. (2013)
		*Bacillus subtilis OTPB1*	*Alternaria solani* and *Phytophthora infestans* responsible for early and late blight	Chowdappa et al. (2013)
		*B. subtilis* FB17	*Pseudomonas syringae* pv. tomato (Pst) DC3000	Lakshmanan et al. (2013)
		*T. harzianum*	Wilt causing pathogen *Rhizoctonia solani*	Manganiello et al. (2018)
		*B. amyloliquefaciens* Ba13	Tomato yellow leaf curl virus disease caused by Tomato yellow leaf curl virus	Guo et al. (2019)
		*Streptomyces pactum*	Tomato yellow leaf curl virus disease caused by Tomato yellow leaf curl virus	Li et al. (2019)
		*Acrophialophora jodhpurensis*	*Rhizoctonia solani* AG4-HGII a fungal pathogen responsible for root rot and crown rot diseases	Daroodi and Taheri (2021)
		*B. atrophaeus* GBSC56	Root-knot nematode *Meloidogyne incognita*	Ayaz et al. (2021)
		*A. jodhpurensis* Msh5	*Alternaria. alternata*, the causal agent of early blight	Daroodi et al. (2022)

**Table 5:** Microorganisms that have exhibited induction of resistance in other horticultural crops.

SN	Crop	Microorganism responsible for IR	Plant disease/pathogen	Reference
1	Cucumber (*Cucumis sativus* L.)	*B. subtilis* M4	*Colletotrichum lagenarium*	Bigirimana et al. (1997)
		*T. harzianum* T-203	—	Yedidia et al. (1999)
		*Streptomyces lydicus* M01	*Alternaria alternata* causing foliar disease of cucumbers	Morcillo et al. (2020)
2	Common Bean (*Phaseolus vulgaris* L.)	*Trichoderma harzianum*	—	Bigirimana et al. (1997)
3	Cucurbits	*B. subtilis* UMAF6639	*Podosphaera fusca* causing powdery mildew	Garcia-Gutierrez et al. (2013)
4	Lettuce (*Lactuca sativa* L.)	*Bacillus amyloliquefaciens* subsp. plantarum	*Rhizoctonia solani* causing bottom rot	Chowdhury et al. (2015)
5	Carnation (*Dianthus caryophyllus* L.)	*P. fluorescens* strain WCS417r	*F. oxysporum* f. sp. dianthi responsible for *Fusarium* wilt disease	Vanpeer et al. (1991)
6	Grapes (*Vitis vinifera* L.)	*P. fluorescens* PTA-CT2	*Plasmopara viticola* and *Botrytis cinerea* causing downy mildew and gray mold diseases of grapes	Lakkis et al. (2019)

**Table 6:** Microorganisms that have exhibited induction of resistance in cereal crops.

SN	Crop	Microorganism responsible for IR	Plant disease/pathogen	Reference
1	Rice (*Oryza sativa* L.)	*Pseudomonas aeruginosa* 7NSK2	Rice blast and sheath blight diseases caused by *Magnaporthe grisea*; *Rhizoctonia solani, Botrytis cinerea*	De Meyer et al. (1999) and De Vleesschauwer and Hoefte (2006)
2	Corn (*Zea mays* L.)	*B. cereus* C1L	*Cochliobolus heterostrophus* soil disease	Huang et al. (2012)
3	Bread Wheat (*Triticum aestivum* L.)	*Trichoderma harzianum*	Spot blotch disease caused by *Bipolaris sorokiniana*	Singh et al. (2019)
4	Barley (*Hordeum vulgare* L.)	*Serendipita vermifera*	Soil borne pathogen *Bipolaris sorokiniana*	Sarkar et al. (2019)

**Table 7:** Microorganisms that have exhibited induction of resistance in plantation crops.

SN	Crop	Microorganism responsible for IR	Plant disease/pathogen	Reference
1	Tobacco (*Nicotiana tabacum* L.)	*B. amyloliquefaciens* FZB42	Leaf blight disease caused by *Phytophthora nicotianae*	Chowdhury et al. (2015) and Wu et al. (2018a,b)
		*Piriformospora indica*	—	Vadassery et al. (2009) and Jogawat et al. (2020)
		*B. cereus* C1L	*Botrytis cinerea* foliar disease	Huang et al. (2012)
2	Tea plant (*Camellia sinensis* L.)	*Bacillus megaterium* DE BABY TRS-4	Brown root rot caused by *Fomes lamaoensis*	Chakraborty et al. (2006)

## 5 Context dependency of MMIR

Based on extensive studies revealing the mechanisms of microbially mediated induced resistance (MMIR), it appears to be a promising strategy for managing pathogens without pesticides. As most of these studies were conducted under highly controlled conditions, their performance under varied field conditions differ due to the effect of biotic and abiotic factors, making them highly context dependent (Diaz et al., 2021). Microbial-mediated IR is found to trigger only under a specific set of environmental factors that affect and change the outcome of microbe-plant interaction, rendering the ISR events unpredictable. For this very reason, the beneficial microbes showing ISR are registered as biostimulants or biofertilizers but not as biopesticides. Therefore, it is necessary to simulate field-like conditions during studies to produce more consistent and predictable ISR technologies. So, it is necessary to understand the effect of various factors on MMIR events.

### 5.1 Biotic factors

Beneficial microbes that are applied in root zone-like PGPRs have to compete for resources and antibiotic production in their interaction with the soil microbiome (Toju et al., 2018), which affects quorum sensing and root-associated biofilm formation of PGPR (Rudrappa et al., 2008a,b). Through this, ISR is activated only when the concentration of beneficial microbes reaches 10^5^–10^7^ colony-forming units (CFU) per gram of root (Bakker et al., 2013). In addition to a successful establishment in the root zone, the induction of resistance also depends on the genetic backgrounds of the plant and microbe. For example, *Pseudomonas putida* strain WCS358r induces resistance in *A. thaliana* (Van Wees et al., 1997), *Pseudomonas fluorescens* WCS374r can induce resistance in radish (*Rhaphanus sativus*) (Leeman et al., 1995), and the *P. fluorescens* strain WCS417r induces resistance in both *Arabidopsis* and radish. Our current understanding of ISR is mostly based on model plants that may not be expressed in crop plants. Therefore, more effort is required to evaluate the strains on crop plants to explore the possibilities of their field applicability.

Similarly, herbivory is reported to induce changes in root exudate profile (Fierer and Jackson, 2006) that affect below-ground microbiota and the colonization of beneficial microbes, thus, affecting the induction of resistance (Gehring and Whitham, 2003; Rudrappa et al., 2008a,b; Yang et al., 2011; Gu et al., 2016; Malacrino et al., 2021). For instance, mycorrhizal colonization is reported to increase, decrease, or remain unaffected by herbivory (Gehring and Bennett, 2009; Barto and Rillig, 2010). Although not studied in depth, some researchers have indicated the possibilities of such alterations in the context of ISR due to insects, parasitoides (Poelman et al., 2011), and facultative endosymbionts found in sucking-type insects like *Hamiltonella defensa* (Su et al., 2015). On the contrary, some researchers have reported a negative effect on chewing insects and positive effect on sucking insects by feeding on the plants with ISR triggered by AMF (Hartley and Gange, 2009; Koricheva et al., 2009). Therefore, it is also required to study the effect of aerial feeding by herbivores on root exudation, the colonization of beneficial microbes, the induction of resistance, and the effect of microbial induction of resistance on herbivory.

### 5.2 Abiotic factors

Microbe-induced resistance is also reported to be influenced by various abiotic factors, including nutrient availability (Miransari, 2013; Oldroyd and Leyser, 2020), soil moisture levels (Auge et al., 2001; Juniper and Abbott, 2006; Ulrich et al., 2019), soil organic matter content (Schnecker et al., 2014; Del Valle et al., 2020), light quality and intensity (Nagata et al., 2015; Konvalinkova and Jansa, 2016), and soil pH (Aciego Pietri and Brookes, 2008). Hiruma et al. (2016) has described the effect of nutrient deficiency on root exudation and ultimately the interaction of plants with beneficial microbes. It is reported that, due to phosphorus (P) deficiency, plants produce strigolactones that play important roles in regulating the interaction of plants with AMF and endophytic fungi (Lopez-Raez et al., 2008; Hiruma et al., 2016). In *A. thaliana*, P-deficiency triggers the expression of PHR1 and PHL1, which are PSR master transcriptional regulators (Hiruma et al., 2016; Castrillo et al., 2017; Morcillo et al., 2020) and results in the induction of JA signaling but the repression of SA signaling. As reported by Khan et al. (2016), the induction of JA signaling resulted in enhanced defense against a leaf-chewing insect in *A. thaliana*, tomato, and tobacco but enhanced susceptibility for an oomycete pathogen and a bacteria (Castrillo et al., 2017). In a classic work from Spagnoletti et al. (2018), it is demonstrated that, due to P-deficiency, soybean plants became 2.5 times more susceptible to charcoal rot disease. However, enhanced AMF colonization resulted in a 5.0-fold induction of resistance. Due to iron (Fe) deficiency, Arabidopsis plants produce defense-related secondary metabolites—coumarins—which affect the rhizospheric microbiota (Stringlis et al., 2018). Due to nitrogen deficiency, the roots of the leguminous plant exude flavanoids to attract rhizobia and trigger their *nod* genes to produce Nod factors (Mbengue et al., 2020). Likewise, most nutrient deficiency is associated with triggered resistance but, in a few cases, adverse effects are also noticed. For example, due to P-deficiency, a recruited PGPR strain, *B. amyloliquefaciens,* induced hypersensitivity in *A. thaliana* by triggering its response to emit a diacetyl volatile compound that caused hypersensitivity (Morcillo et al., 2020). Here, it is noteworthy that availability of nutrients and plant defense activation is directly related. This should be explored and standardized for exploiting ISR under field conditions instead of using costly defense metabolites that are difficult to synthesize in sufficient amounts for field application (Gershenzon, 1994; Neilson et al., 2013).

### 5.3 Interplay between biotic and abiotic factors

ISR triggered by PGPRs is regulated by phytoharmones like JA, ET, SA, and other members of the oxylipins family (Pieterse et al., 2014; Vlot et al., 2020) and by activation of a network of signaling molecules including reactive oxygen species (ROS) (Camejo et al., 2016) and reactive nitrogen species (RNS) (Khan et al., 2019). Plants are smart enough to integrate information about their biotic and abiotic environment, resulting in cross-talk between different signaling pathways (Fujita et al., 2006; Rejeb et al., 2014). This capacity helps them to understand the challenges they are facing and to prioritize and fine-tune their responses to that (Robert-Seilaniantz et al., 2011). This means the presence of ISR potent microbes in soil is not sufficient to trigger ISR but their interplay with other biotic and abiotic factors in their environment can activate an ISR response in plants (Pozo et al., 2015) under any given situation. Therefore, it is necessary to study how responses to other biotic and abiotic factors integrates with the phytohormonal system to trigger MMIR. For example, nutrient deficiencies are found to alter the root exudation patterns, thus affecting the chemotactic responses for selective accumulation of beneficial microbes in the rhizosphere for triggering ISR (Hiruma, 2019). Therefore, it is imperative to conduct studies to understand the interplay of biotic and abiotic factors for sustainable field response of MMIR before opting for commercial application.

### 5.4 Agricultural practices

Regular farm practices like tillage, fertilization, and pest management greatly affect soil micro-biome and plant microbe interactions (Banerjee et al., 2019; Caradonia et al., 2019). In horticultural crops, beneficial microbes are added to growth media or substrate (that is not soil) where microbial inoculants face less competition. For field crops, microbial inoculants are added to farm soil, where they face much heterogeneous competition. Interestingly, on-farm crops are typically grown under well-fertilized conditions to achieve higher yields, which contrasts with the low-nutrient conditions that favor colonization by beneficial microbes and the induction of resistance. For instance, due to long-term phosphate fertilization, percent root colonization in maize by AMF was reported to be reduced (Wang et al., 2017) and, due to P supplementation (Gange et al., 1999) and Nitrogen supplementation (Gange and Nice, 1997), resistance to chewing-type insects was found to be reduced. Contradictory results were recorded by Vesterlund et al. (2011) when fertilization was found to improve the performance of fungal endophytes against chewing-type insects. Thus, controlled fertilization can be potentially used to selectively recruit beneficial microbes (Bakker et al., 2018; Oldroyd and Leyser, 2020). Apart from fertilization, other farm practices like tillage and crop rotation also have profound effects on microbial populations. Due to long-term organic farming, beneficial microbes become established in soil and induce resistance in subsequently grown crops (Pineda et al., 2020). Tillage can reduce the build up of insect pests in soil but also disturb the establishment of beneficial microbes and creation of disease-and pest-suppressive soils (Peters et al., 2003).

## 6 Future strategies to increase the use of microbes inducing resistance

From the above discussion, we now understand that inconsistent expression of microbial-mediated induced resistance is the underlying fact that needs to be addressed to utilize this technology. Therefore, to handle the context dependency of microbial-mediated IR, it is required to select beneficial microbes after screening large numbers of microbial strains tested for ISR activation across the varied climatic conditions. It is required to optimize agronomic practices to provide favorable field conditions for consistent expression of ISR by established beneficial microbes. For example, optimization of phosphate fertilization would be useful for specific strains of beneficial microbes. In this context, it is also important to standardize the formulation, composition, and method of application of bioinoculants. Therefore, efforts are underway (Vassilev et al., 2020) to develop formulations that are least affected by the environment like gels, encapsulation, and seed coating. Scientists are also trying to develop a consortium of many species rather than single species (Vallad and Goodman, 2004; Bradacova et al., 2019). A consortia of many effective strains of similar species of PGPR collected from different locations in a specific agro-climatic zone should be prepared for that particular zone. Mode of application and doses are also being standardized. As per the opinion of Mitter et al. (2019), the development of more responsive plant genotypes can help to improve the consistency of microbial-mediated IR. This would involve having root exudates to increase the supply of beneficial microbes, enable better symbiosis and enhanced plant responses towards symbiosis, and, in turn, enhanced induction of resistance to tackle diseases and insect pests, as explained by Tetard-Jones et al. (2012) and Hohmann et al. (2020). For example, advances include the development of transgenic varieties carrying the NPR1 gene from Arabidopsis (Cao et al., 1997); identification of new genes, such as those involved in bacterial recruitment and plant defense independent of malate efflux, revealed through root transcriptome analysis of *Arabidopsis thaliana* exposed to beneficial *Bacillus subtilis* FB17 rhizobacteria (Lakshmanan et al., 2013); sequencing of LysM-type receptor kinase genes involved in legume perception of rhizobial signals (Madsen et al., 2003); and the development of near-isogenic lines of various crops incorporating the NPR1 gene and other PR genes into high-yielding genetic backgrounds. Screening of available germplasms could enable higher root exudation of 7,4′-dihydroxyflavone and naringenin exudates and a greater presence of chemotaxis (Szoboszlay et al., 2016). Alternatively, as suggested by Diaz et al. (2021), we may accept that it is not possible to generalize the package of practices to support ISR events in all types of plant-microbe interactions. Another approach that is being studied by Compant et al. (2019), Wang and Li (2019), Arif et al. (2020), and French et al. (2021) advocates for completely controlling the microfauna in rhizosphere. This can be achieved by rotation of crops (Latz et al., 2016; Pineda et al., 2017; Veen et al., 2019; Pineda et al., 2020) to support sufficient build up of beneficial microbes and by soil amendments (Shen et al., 2019), so as to make soil suppressive to pests and diseases. For example, phosphate fertilization should be standardized to reduce charcoal rot of Soybean (Spagnoletti et al., 2018) as for the development of disease-suppressive soils through crop rotation and tillage management practices (Peters et al., 2003). Today, our understanding of ISR is mostly based on model plants and may differ for crop plants. Therefore, more effort is required to evaluate the strains on crop plants to explore the possibilities of their field applicability. Future attempts to unravel more detailed regulatory mechanisms on transcription factors involved in beneficial microorganism-mediated ISR will improve our understanding of the formation and regulation of ISR. Therefore, it is also required to study the effect of aerial feeding by herbivores on root exudation, the colonization of beneficial microbes, and the induction of resistance and effect of microbial induction of resistance on herbivores and their natural enemies. If we accept the context dependency of microbial-mediated IR is inevitable, we need to focus on developing environmentally stable commercial formulations and compositions of secondary metabolites of beneficial microbes that are known to trigger the resistance in laboratory conditions, as they may induce resistance consistently under field conditions as well. This will require more interdisciplinary research and partnership with industries.

## 7 Conclusion

Despite more than 25 years of research, the commercial application of cross-protection through induced resistance has not become a reality, largely due to the gap between laboratory results and field applications, where the effects of MMIR with known biostimulants are often diminished by biotic and abiotic factors as well as field conditions. Due to the inconsistent induction of resistance, these beneficial microbes are merely acknowledged for their ability as growth promoters or biostimulants and not for their biocontrol potential. To improve the field applicability of MMIR, it is essentially required to get consistent results under a given set of conditions. This requires more experimentation that simulates field-like conditions in a laboratory setting. Even with this, standardization or identification of some common practices to support consistent triggering of MMIR in all plant-microbe interactions under all field conditions seems impractical. Instead, incorporating NPR1 genes and other PR-genes in crop plants and transferring them to high-yielding genetic backgrounds seems to be the most exciting and promising area for future research in MMIR. Similarly, standardization of phosphate fertilization to enable host interaction with beneficial microbes may improve the field stability of MMIR. Mass production of resistance triggering secondary metabolites of beneficial microbes and preparation of their stable commercial formulations for field applications through the collaboration of researchers, industries, and policy makers may produce a widely applicable technology.

## Glossary

ABA: Abscisic Acid
AMFs: Arbuscular Mycorrhizal Fungi
APX: Ascorbate Peroxidase
BAK1: BRI1-Associated Kinase
bHLH: Basic Helix Loop Helix transcription factor
BIK1: *Botrytis*-Induced Kinase1
BKK1: BAK1 LIKE1
Ca^2+^: Calcium ion
CALS10: Callose Synthase gene
cAMP: Cyclic Adenosine Monophosphate
CAT: Catalase
CDPK: Calmodulin-Dependent Protein Kinase
CERK1: Chitin Elicitor Receptor Kinase 1
CFU: Colony-Forming Units
Cl^−^: Chloride ion
CNGC19: Cyclic Nucleotide Gated Channel 19
DEGs: Differentially Expressed Genes
EDS1: Enhanced Disease Susceptibility 1 protein
EPS: Extracellular Polysaccharides
ERF1: Ethylene Response Factor1
ET: Ethylene
ETI: Effector-Triggered Immunity
Fe: Iron
FLS2: Flagellin Sensing 2 receptor
GGAT1: Glutamate Glyoxylate Amino Transferase
GLR 2.4: Glutamate Receptor 2.4
GLR 2.5: Glutamate Receptor 2.5
GLR 3.3: Glutamate Receptor 3.3
GPX: Glutathione Peroxidase
H^+^: Hydrogen ion
H_2_O_2_: Hydrogen Peroxide
IAA: Indole 3 Acetic Acid
IR: Induced Resistance
ISR: Induced Systemic Resistance
ITS: Internal Transcribed Spacer
JA: Jasmonic Acid
K^+^: Potassium ion
L-MA: L malic acid
LOX: Lipoxygenase
*LOX1*: Lipoxygenase encoding gene
LPS: Lipopolysaccharides
LysM: Lysin Motif receptor-like kinase
MAMPs: Microbial Associated Molecular Patterns
MAP Kinase: Mitogen Activated Protein Kinase
miR825: Micro RNA 825
MMIR: Microbial-Mediated Induced Resistance
MVCs: Microbial Volatile Compounds
MYB: v-myb avian myeloblastosis viral oncogene homolog
MYB72: MYB domain protein 72 transcription factor
MYC2: Myelocytomatosis oncogene 2
N terminal: Amino terminus
NB-LRR: Nucleotide Binding Leucine Rich Repeat
Nod factors: Nodulation factors
*NPR1*: Nonexpressor of Pathogenesis-Related genes 1
O^2−^: Superoxide anion
OH ^−^: Hydroxyl radical
OST1: Open Stomata 1
P: Phosphorus
PAMPs: Pathogen-Associated Molecular Patterns
PDF1.2: Plant Defensin 1.2
PDLP5: Plasmodesmata-Located Protein 5
*PEN2*: Penetration 2 gene
*PEN3*: Penetration 3 gene
PGP: Plant Growth Promoter
PGPR: Plant Growth-Promoting Rhizobacteria
PHL1: PHR 1 Like1
PHR1: Phosphate Starvation Response 1
POX: Peroxidase
PP2C: Type 2C Protein Phosphatases
PPO: Polyphenol Oxidase
*PR* genes: Pathogenesis-Related genes
PR proteins: Pathogenesis-Related proteins
PRRs: Pattern Recognition Receptors
PSR: Phosphate Starvation Response
PTI: Pattern Triggered Immunity
RNA: Ribo Nucleic Acid
ROS: Reactive Oxygen Species
SA: Salicylic Acid
SAR: Systemic Acquired Resistance
SLAC1: Slow Anion Channel-Associated1
SnRK2: SNF 1 Related Protein Kinase 2
SOD: Superoxide Dismutase
tub2: Beta tubulin2
VOCs: Volatile Organic Compounds
